# Epistemic Network Analyses of Economics Students’ Graph Understanding: An Eye-Tracking Study

**DOI:** 10.3390/s20236908

**Published:** 2020-12-03

**Authors:** Sebastian Brückner, Jan Schneider, Olga Zlatkin-Troitschanskaia, Hendrik Drachsler

**Affiliations:** 1 Department of Business and Economics Education, Johannes Gutenberg-University Mainz, 55128 Mainz, Germany; Troitschanskaia@uni-mainz.de; 2 Information Center for Education, DIPF Leibniz Institute for Research and Information in Education, 60323 Frankfurt Am Main, Germany; drachsler@dipf.de; 3 Computer Science Faculty, Goethe University, 60323 Frankfurt am Main, Germany; 4 Educational Science Faculty, Open University of the Netherlands, 6419 AT Heerlen, The Netherlands

**Keywords:** epistemic network analysis, eye-tracking, graph understanding, economics, higher education

## Abstract

Learning to solve graph tasks is one of the key prerequisites of acquiring domain-specific knowledge in most study domains. Analyses of graph understanding often use eye-tracking and focus on analyzing how much time students spend gazing at particular areas of a graph—Areas of Interest (AOIs). To gain a deeper insight into students’ task-solving process, we argue that the gaze shifts between students’ fixations on different AOIs (so-termed transitions) also need to be included in holistic analyses of graph understanding that consider the importance of transitions for the task-solving process. Thus, we introduced Epistemic Network Analysis (ENA) as a novel approach to analyze eye-tracking data of 23 university students who solved eight multiple-choice graph tasks in physics and economics. ENA is a method for quantifying, visualizing, and interpreting network data allowing a weighted analysis of the gaze patterns of both correct and incorrect graph task solvers considering the interrelations between fixations and transitions. After an analysis of the differences in the number of fixations and the number of single transitions between correct and incorrect solvers, we conducted an ENA for each task. We demonstrate that an isolated analysis of fixations and transitions provides only a limited insight into graph solving behavior. In contrast, ENA identifies differences between the gaze patterns of students who solved the graph tasks correctly and incorrectly across the multiple graph tasks. For instance, incorrect solvers shifted their gaze from the graph to the *x*-axis and from the question to the graph comparatively more often than correct solvers. The results indicate that incorrect solvers often have problems transferring textual information into graphical information and rely more on partly irrelevant parts of a graph. Finally, we discuss how the findings can be used to design experimental studies and for innovative instructional procedures in higher education.

## 1. Introduction

Understanding graphs has been considered an essential skill both in everyday life (e.g., understanding newspapers) and in most academic disciplines [1,2,3]. Graphs serve to simplify the representation of complex concepts and can facilitate the development of conceptual knowledge in a domain [4,5,6,7]. 

Line graphs are a graph form that is used particularly frequently in various domains and has been intensively researched for years [1,8]. Phenomena from several disciplines, such as the relationships between time and velocity in physics or between time and costs in finance, can be visualized similarly in line graphs due to the mathematical conceptualization of graphs as well as basic conventions for their representation (e.g., axes, axis labels, linear progressions, function-based representations) [3,9,10]. Often, the graphs are embedded in text-based instruction to aid the perception of textual description and to supplement it by presenting a learner with further information in the compressed form [11].

With the development of standardized tests for graph understanding (e.g., the Test of Understanding Graphs in Kinematics (TUG-K) [8], the systematic assessment of graph understanding based on test scores was intensified in physics education research [12,13,14,15]. Beyond science, there are various domains, for instance, finance and economics, that frequently use graphs to illustrate complex concepts and models and make them more comprehensible [9,11,16]. For example, to understand changes in profit, cost, market share over time or to analyze share price developments and inflation, a fundamental knowledge of how graphs are constructed and what the lines, labels, and axes mean is required. In economics education, they are therefore considered already at the beginning of economics studies [17,18]. 

Despite the high importance of line graphs in economics and finance contexts, there is little research on students’ graph understanding in this domain so far. The few existing studies address merely the instructional quality of financial graphs in textbooks [16], the construction principles of graphs [11], and potential factors influencing the use of graphs in instruction [19]. Whether the comprehensive findings on graph understanding, in particular from physics education research, can be transferred and applied in the context of economics and finance is under-researched [20]. Previous studies focusing on the contexts of economics and physics indicated differences in graph understanding at the level of individual tasks concerning the graph concepts ‘area under a curve’ and ‘slope of a curve’ and the task’s level of mathematical requirements [1,3,20]. Since the transfer of graph understanding between contexts seems to be difficult [21], there is a need for more studies that examine how the knowledge and skills acquired in one domain are applied to graph tasks from different domains in a comparative way. 

Due to technological developments, a large number of recent studies, especially in the natural sciences, used eye-tracking to observe the process of graph understanding [15,22,23]. Current eye-tracking studies mainly examine students’ dwell time, frequency or duration of fixations on individual graph components [1,3,20]. These analyses, however, reflect only an isolated part of students’ graph task solution behavior. The extent to which a graph task with its different components such as axes, curves, and labels has been solved only becomes evident when examining how the students integrate and process the information presented in the whole task. The transitions between looking at a task’s instructional text and the individual components of a graph included in the task (gaze behavior) during the response process therefore need to be analyzed to gain insights into the ‘quality’ of students’ graph understanding.

To address this research deficit, we build on an earlier eye-tracking study [1] (see Section 3.1) with 23 economics students solving eight-line graph tasks in finance and physics contexts. We observed the students’ gaze fixations and transitions while they were solving the tasks to gain an understanding of their transitional gaze behavior. In this study, we argue that students’ gaze behavior while solving graph tasks provides insights about the quality of students’ graph understanding, which manifests in the final correct or incorrect task response. Accordingly, we analyzed the fixation-based transitions to compare response processes that lead to correct and incorrect responses. 

For a holistic analysis of the response process using transitions, we use epistemic network analysis (ENA), a novel approach from the field of learning analytics [24,25,26]. Using this approach, the transition patterns corresponding to the response processes of correct and incorrect solvers can be visualized and compared. In this study, we illustrate that ENA can consider the complexity of eye movements during a response process and identify general differences between eye movements depending on the students’ test scores.

## 2. Conceptual and Methodological Background

### 2.1. Response Processes of Solving Graph Tasks

There are various theories of graph understanding [5,6,27,28]. Though some theories only focus on the understanding of graphs without texts, understanding graph tasks usually requires text-image integration [29]. In graph tasks, a text-based explanation of the concepts or facts presented in the graph is usually included and must be processed by a learner. Solving graph tasks therefore require models that relate both texts and graphical representations. To understand and explain graph solving behavior and strategies, integrative models of text and visual comprehension, so-termed “models of multimedia learning”, were established [30,31]. 

Since graph tasks usually first present an introductory question textually and then learners have to examine the graph with regard to the question to develop a solution, the integrative models are especially important for the description of mental processes in graph understanding. When solving graph tasks, learners mostly generate situational models of text content and its structure at first [31]. This involves reciprocal encoding and decoding processes in which the relevant textual features of the graph task are recognized and given meaning. After task solvers have developed a situational model of the problem presented, they turn their attention to the graph. It is assumed that the graph reader first mentally decodes various components of the graph (slope, axes, course, labels), assigns them a meaning and relates them to each other in the context of their internal mental representation of the concept in question. Here, graphical elements (graph sequences, colors and shapes, axis representations) need to be connected to each other to create meaningful contexts and to construct a coherent mental model to enable an adequate interpretation of a graph. 

If scheme-driven graph understanding processes are additionally activated, mental models are developed that can connect the created internal representations of graphs and text on the basis of semantic relations [32]. Since the “internal depictive representations” are developed by learners on the basis of both textual and graphical representations, i.e., perceptual and conceptual cognitive processes [31], after reading and understanding a question, graphs can be explored systematically. Particularly, axis tick labels and graph labels can be related to the axis ticks and slopes of a graph, understood and analyzed in the context of a textual question [1,2]. In this study, therefore, we follow the established integrative models of graph understanding when conceptually describing response processes of solving graph tasks [31]. 

### 2.2. Eye-Tracking Research on Students’ Difficulties in Graph Understanding

Understanding graphical representations are generally challenging for students [8,14,21]. The difficulty that students often experience when first encountering graph tasks depends on the graphically represented concepts. Reading a point from a line graph with its coordinates, for example, presents a different challenge than interpolating between several data points or even predicting graph trends and extrapolation beyond the representation of a graph [4]. Especially in the field of physics education research, the determination of the slope of a graph vs. the area under a graph and the degree of mathematical requirements have been identified as determinants of the level of task difficulty [13,22,33].

In recent studies, eye-tracking was used to get a closer look at students’ response processes [1,3,20] to get an indication of the difficulties of graph understanding. This method enables the analysis of not only the test scores but allows researchers to determine with which frequency and sequence a learner looks at individual parts of a line graph—so-termed Areas of Interest (AOIs)—during the process of task solving (e.g., axis labels, axis tick labels, response options like the attractor or distractor, the curve). The frequency is often operationalized by the number of fixations that occur within an AOI. Fixations are stable gaze patterns in which the eye is directed at a fixed spot and this gaze is maintained for a certain period [34]. The gaze shifts between the fixations, which also represent the sequence of focusing, are termed saccades. They mark which areas a learner looks at one after the other. Both types of eye-tracking metrics are used as indicators of the learner’s attention [35] and the cognitive processes that take place while doing so (according to the eye-mind assumption [34]). For instance, fixation duration, cumulative fixation duration, visit duration, fixation frequencies are often chosen as indicators of cognitive effort [36,37].

Studies that examined the understanding of line graphs in physics and economics contexts considered the dwell times and fixation times on AOIs of a graph and suggested that certain eye-tracking metrics provide initial indications of students’ learning difficulties [1,20]. Relevant AOIs (i.e., areas containing critical information, e.g., axes) were viewed longer and with higher frequency by physics students who show a higher understanding of graphs [1,3]. Compared to physics students, economics students showed a lower overall graph comprehension on average—especially for ‘area under the curve’ tasks—, which was reflected in lower test scores but also in a longer dwell time [1,20]. A dedicated research effort on the differences in gaze patterns during the response process of individual graph tasks that goes beyond the dwell times is, therefore, necessary to investigate such differences concerning certain task types and contexts.

### 2.3. Transitions between AOIs as a Predictor of the Quality of Graph Understanding

Some studies conducted more fine-grained analyses and examined the eye movements that learners make between individual pairs of AOIs of a graph task. In eye-tracking studies, these gaze shifts between AOIs are called transitions, which represent an eye-tracking metric that indicates the mental relations a learner tries to construct between different parts of a graph task [38,39]. These transitions are saccades between AOIs [29] and the transition frequency is “the number of gaze shifts between AOIs” [37]. Since a task solver needs to integrate different information from axes, texts, labels to be able to interpret a graph appropriately, an analysis of transitions constitutes a significant measure for describing response processes when solving graph tasks [29,40]. Individual transitions between textual or verbal representations of information and graphs have therefore often been used as a significant predictor of the quality of understanding processes [24,41].

Analyses of eye-tracking data often also include analyses of transitions [34,42]. Especially in spatial and graphical representational contexts, they are often used to analyze the understanding of different graphical elements in relation to each other (e.g., for analyzing geovisualization tools). In other studies, focused on analyzing response processes on multiple-choice tests, transitions are also frequently examined [23,43]. However, only a few studies explicitly focus on transition analysis on kinematic line graphs [39,41,44,45]. A study by Mason et al. [39] on graph-text comprehension indicated that learning success is higher for those students who try to integrate textual and visual representations more often, i.e., where there are more transitions between relevant texts and graphs for correct responses. Jian et al. [44] and Smith et al. [45] pointed out that transitions occur frequently, especially in adjacent areas, and that learners often try to combine abstract and textual representations. In a study on the interpretation of vector fields, Klein et al. [41] used graphs as explanatory aids and demonstrated that more frequent transitions between textual and graphical representations promote the understanding of vectors. Studies on solving tasks related to the electrical circuit also showed that learners with correct responses more often returned from the task to the problem description [40]. In a study by Tsai et al. [23], predictions of debris slide hazards were graphically visualized and students were asked to identify the weather conditions that lead to them. Students who gave incorrect answers had significantly more transitions between the graphical representation of the slope and the problem statement.

In these studies, however, only isolated transitions were analyzed so far. For example, transitions between AOIs of the graph or between parts of the graph and an instruction text were always evaluated separately concerning differences in learning success (e.g., experts vs. novices). Since fixations are sequential and often limited to certain parts of the task, however, the participants’ gaze behavior while solving the whole task needs to be considered [44,45]. For example, a respondent can move their gaze back and forth between a curve and an axis intercept more often simply because they also frequently look at the curve, and such areas are adjacent. At the same time, a longer dwell time on the curve indicates that the respondent will be less likely to move their gaze back and forth between individual response options. The interpretation of a transition must generally be evaluated against the occurrence of the other transitions in a response process. 

Epistemic network analysis (ENA, see Section 3.6) allows for the modeling of data structures, whereby the transition structure is the most important aspect of the analysis [24,25,26]. In the case of the analysis of eye-tracking data, transitions between AOIs can be interpreted as connections. ENA creates a network structure based on these connections and allows for a comparison of groups. Concerning graph understanding, comparisons can be made between students who understand graph tasks and students who do not. Therefore, we argue that in the case of eye-tracking data for graph tasks, ENA can contribute to a holistic analysis of the transition structure between AOIs while offering insights into the participants’ understanding or lack of understanding of graphs.

### 2.4. Research Questions

The aim of the study is to investigate how eye-tracking can be used to provide insights into graph understanding. The present study ties in with the outlined research gap by examining the differences in eye fixations and transition patterns of first-year economics students solving tasks on graph understanding. To operationalize the identification of graph understanding, in this study, we identify the correct solution to a graph task as the understanding of it. Concerning the fixations this study examines the proportion of fixations on different AOIs in contrast to their frequency because everybody takes a different number of fixations to solve the tasks. To guide our study, we focus on the following research questions (RQ):

Our first RQ, concerns with the recognition of graph understanding based on fixation patterns: 

RQ1: How do students who solved a task correctly (correct solvers) differ from students who solved a task incorrectly (incorrect solvers) in their proportion of fixations on different AOIs of the task graphs?

RQ2: How do correct and incorrect solvers differ in their transition patterns between different AOIs of the task graphs?

To answer these RQs, in this study, we tracked the students’ gaze while solving eight linear graph tasks.

## 3. Method

### 3.1. Background Information

To conduct the study, we used a modified subset of the data collected by Klein et al. [1] and applied an ENA approach to analyze the central transitive and fixation-specific features. The similarities and differences between our study and the study by Klein et al. [1] are described in the following sections. The commonalities include the use of the same tasks and assessment design. The differences include the sample used, the definition of AOIs, the data extraction, and the analytical focus on fixation frequencies and transitions.

### 3.2. Commonalities

#### 3.2.1. Tasks

**Figure 1:**
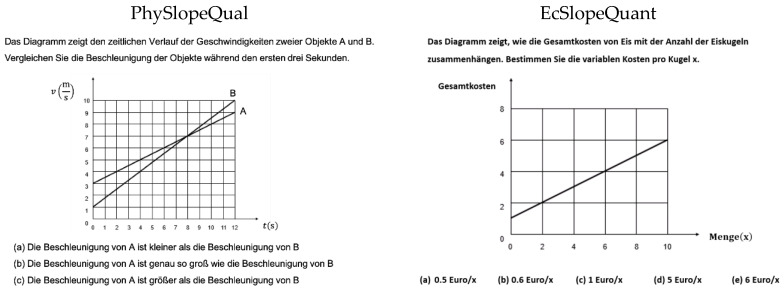
Original tasks PhySlopeQual and EcSlopeQuant solved by the participants.

**Figure 2:**
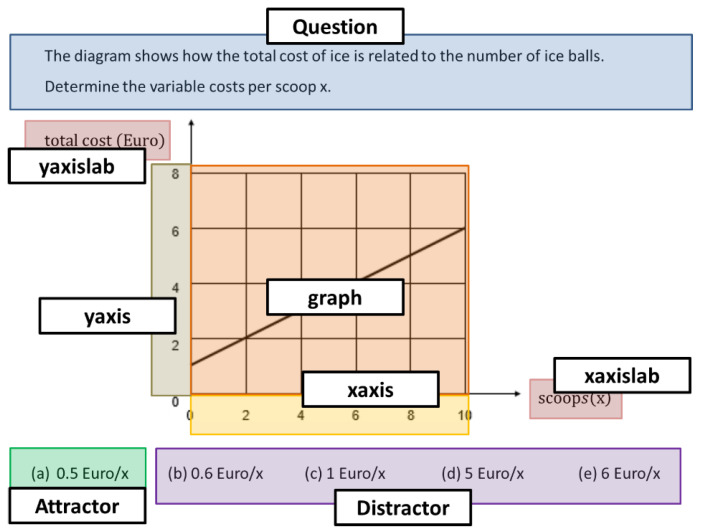
AOIs of a single graph task.

The data used in both this study and the study by Klein et al. [1] was collected to assess graph understanding. In the present study, we used the same four isomorphic pairs of linear graph tasks (four economics and four physics tasks) the participants had to solve in the study by Klein et al. [1]. All eight tasks have an identical structure. The task prompt was always presented first. Then, a graph with one or two curves was presented, followed by a number of different response options, and the participants were required to choose one. The tasks included graphical concepts (here: interpretation and calculation of the slope of a curve and area under a curve) that are taught systematically in secondary school mathematics courses. Thus, participants should already have the mathematical knowledge needed to solve them. To explore whether previous knowledge on the topic of a graph task affects how participants understand and solve that graph task, we decided to focus on the topics of physics and economics. The topic of physics was selected because it is a mandatory topic in secondary education curricula in all German federal states and all participants learned about this topic in school. In contrast, the topic of economics is not systematically taught in secondary education in Germany [46], which means most participants did not receive any significant formal training on that topic. Thus, we used both domains to be able to conduct a comprehensive analysis on graph understanding in domains the students were either well familiar or less familiar with. In this paper, we present the figures of two sample tasks, as shown in Figure 1 and Figure 2 (due to the copyright issues, we have illustrated only two different tasks in Figure 1 and Figure 2 as examples). Figure 1 presents the original tasks PhySlopeQual and EcSlopeQuant in German and Figure 2 shows an English translation of the task EcSlopeQuant.

The graph in the second economics task (EcAreaQuant) displays a linear function representing how wages increase based on the working hours. Participants have to select the earnings of an employee after working for a defined number of hours. The second physics task (PhyAreaQuant) also displays a linear function, this time representing the speed of a car in relation to time. Participants have to select how far the car moved in a certain amount of time. 

The graph in the third economics task (EcSlopeQual), displays two linear functions representing the price trend for two different shares. Participants have to compare the growth rate of the stocks at a particular time. The graph of the third physics task (PhySlopeQual) displays two linear functions that represent the velocity of two objects over time. Participants have to select which object has the greater acceleration. 

Finally, the graph of the fourth economics task (EcSlopeQuant) shows a linear function representing the cost of a portion of ice cream in relation to the number of scoops (see Figure 1 and Figure 2) [1]. Participants have to determine the variable cost per scoop. The fourth physics task (PhySlopeQuant) shows a linear function representing the speed of a train over time. Participants have to determine the acceleration of the train.

#### 3.2.2. Procedure and Apparatus

**Figure A1:**
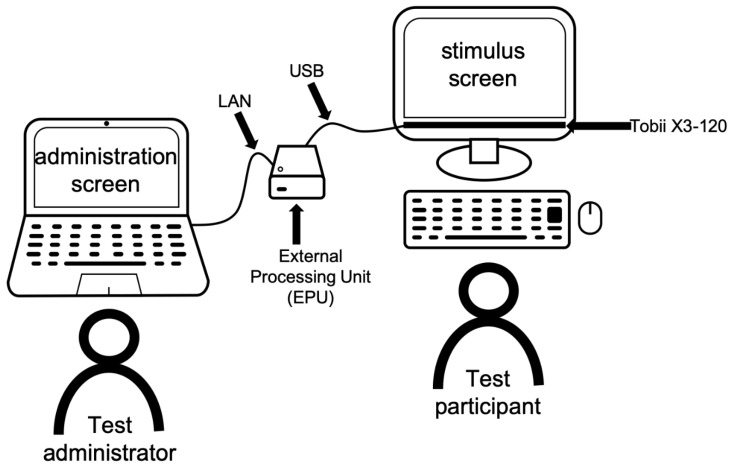
Experimental Setting.

Another commonality of the two studies is the identical assessment design. Each study participant had to consecutively solve four graph-based economics problems and four graph-based physics problems while their eye movements were recorded. To record eye movements, we used Tobii X3-120 eye-trackers. The eye-tracker was placed underneath a 22-inch monitor with a resolution of 1920 × 1080 pixels and the participants sat, equipped with a mouse for clicking on the selected responses and a keyboard for navigating the test, at an average distance of 60 cm from the screen (see Appendix A Figure A1 [1]). The eye-trackers recorded 120 frames per second, and the aforementioned setup allowed the participants to move their heads relatively freely without impairment.

After an introduction to the technology and the setting, a 9-point calibration was performed with each participant. The tasks were then presented to the participants in a randomized order, with the condition that no two isomorphic tasks were ever presented in succession. After each task, which consisted of the task question, the graph and the response options (Figure 1), only the response options were presented again and the students were asked to select one of four response options with a mouse click; the configuration was done with a HTML radio button syntax. By pressing the spacebar, the students could determine the progress of the experiment on their own. 

After the eye-tracking experiment, the participants filled out an additional paper-pencil questionnaire to assess their sociodemographic characteristics (e.g., gender, age, the school type attended for secondary education, the family language and educational background of the parents).

### 3.3. Differences and Extensions

In the present study, the central difference compared to the study in Klein et al. [1] lies in the analytical approach. The first difference is already subject to the AOI specification. While Klein et al. [1] did not differentiate between the *y*-axis and *x*-axis and the corresponding labels, for this study different AOIs were specified for each axis and each label. Furthermore, in this study, the AOI graph refers only to the curve and the surrounding plotting area and does not additionally include the axes. These are indicated separately. Besides, a distinction is made between attractors and distractors, while Klein et al. [1] treated the entire multiple-choice option in an aggregated manner. The exact representations of the AOIs are shown exemplarily in Section 3.3.

A second difference lies in the analysis we used. While Klein et al. [1] compared the dwell times and gaze shifts in the AOI graph between students from different domains, the present article focuses on the frequencies and percentages of fixations of first-year economics students on newly specified AOIs. Of particular interest are differences in fixation frequencies between correct and incorrect solutions and analysis of transitions between AOIs (see Section 3.4). Since the transitions are also considered globally, the ENA complements a method that has hardly been used for the analysis of eye-tracking data (see Section 3.5)

### 3.4. Participants

For this study, we used the data of 23 (15 female) first-semester students of economics from one German University. The average age of the participants was 20.07 (1.86) years. This was slightly lower than the average age of 20.52 (2.75) found for the Germany-wide representative sample of 7,679 first-year economics students in the WiWiKom study [47]. The average grade of university entrance qualification in the sample was 2.30 (4.05), which was slightly below the average of 2.32 (5.84) of the WiWiKom sample [48]. A short test of the German version of the Test of Economics Literacy (TEL-4) was used to assess previous domain-related knowledge. A comparison with the results of the WiWiKom sample showed that the sample used here has, Spearman-Brown corrected, comparable knowledge (Mean = 8.472) [49] to the mean of the 23 students (Mean = 9.217). The students received 10 Euros for their voluntary participation in this study, which lasted about 45 min.

### 3.5. AOIs

To determine the number and sequence of fixations for each AOI, we used an Identification of Velocity Threshold (I-VT) algorithm [50]. Only fixations that lasted at least 100 milliseconds were considered to ensure that only fixations are used that reflect the students’ awareness and are not part of mainly physical, microsaccadic fixations [37]. In comparison to previous studies [1,3], we extended the definition of AOIs for a graph task in differentiating between the *x*-axis and the *y*-axis as well as the attractor and the distractors, as these parts are relevant for correct and incorrect solutions. Thus, each graph task presented the following AOIs (see the rectangles in Figure 2) that are relevant for solving the tasks:

- AOI_Question represents the area of the screen where the task question was displayed.
- AOI_yaxislab represents the area of the screen where the label for the *y*-axis was displayed.
- AOI_xaxislab represents the area of the screen where the *x*-axis label was displayed.
- AOI_graph represents the area of the screen where the graph was displayed.
- AOI_Attractor represents the area of the screen where the correct answer (attractor) was displayed.
- AOI_yaxis represents the area of the screen where the *y*-axis was displayed.
- AOI_xaxis represents the area of the screen where the *x*-axis was displayed.
- AOI_Distractor represents the areas of the screen where the incorrect answers (distractors) were displayed.

### 3.6. Data and Analysis

The structure of the dataset is determined by the students’ time-sequential task response processes, i.e., at least every 100 ms. A dichotomous score indicates whether a fixation occurs within an AOI of a task (=1) or not (=0). In total, the dataset contains 184 response processes (23 students × 8 tasks) and 37,411 fixations. Each line (see Table 1) describes a specific fixation and its affiliation (i) to a case, which is listed in the index for the 184 response processes, (ii) to a participant’s alphanumeric seven-digit code, which was individually generated at the beginning of the test, (iii) to a media, which refers to a specific task and (iv) to socio-biographical variables (only gender and age are presented here). For each fixation, the recording time in milliseconds (including the start and the end of a fixation), the fixation index, which indicates the number of a specific fixation among all fixations of a respondent, and the several AOI variables a fixation belongs to are shown. The dichotomous scoring for the AOI variables indicates whether a respondent looked at an AOI at a specific point in time (Table 1).

To identify and count the number of transitions, first, a string variable was generated that included the AOI at which the respondent was looking at a specific point in time. Secondly, successive identical AOIs were deleted. Two of each of the remaining AOIs now indicated whether a transition occurred and if so, what type of transition it was. For example, two transitions can be found in the in the sample data shown in Table 1: The first transition, after the start of the response process for this task, begins at the AOI Question (fixation index 1114) and ends at the AOI graph (fixation index 1115). The second transition starts at the AOI graph (fixation index 1116) and ends at the AOI Question (fixation index 1117). While solving the eight tasks, the 23 participants transitioned their gazes among the different AOIs 9501 times. 

In Table 2, the number of transitions in which an AOI is involved are noted for each AOI. If, for example, a respondent shifts his gaze from the AOI_Question to the AOI_graph and back again, this is counted as two transitions for the AOI_Question and two transitions for the AOI_graph. If the respondent turns from the AOI_Question to the AOI_yaxislab and back to the AOI_graph, then AOI_Question now has three transitions, AOI_yaxislab two and AOI_graph also three. According to this calculation scheme, 1328 transitions are assigned to the AOI_Question and 6.987 to the AOI_graph when all response processes are considered. AOI_graph was even part of most of the transitions and the AOI_xaxislab was least involved in transitions (793 times). 

We argue that transitions among the AOIs do not happen randomly. Cognitive processes while students solve the tasks are responsible for triggering these transitions. We therefore consider that there are some transitions between AOI_Question, AOI_graph and others that provide insights into the graph task solving strategy applied by a participant. To analyze the importance of the transitions between the AOIs of students’ fixations for solving the graph tasks, we consider both the individual transitions as they occur among correct and incorrect solvers and the whole graph solving process. Thus, we take a holistic view of the graph task solving process, since it is assumed that transitions influence each other and occur with different frequencies in the solving process. For instance, transitions occur more frequently between adjacent AOIs [44], i.e., more transitions between AOIs (e.g., between graph and yaxislab) can cause more transitions to adjacent AOIs (e.g., between yaxis and yaxislab) than to distal AOIs (e.g., between question and xaxislab). To consider these interrelations of transitions, we applied Epistemic Network Analysis (ENA) [24,25,26] to our data using the ENA Web Tool 1.6.0 [51].

### 3.7. Epistemic Network Analysis (ENA)

ENA is a quantitative ethnographic technique that enables modelling structured connections of data. ENA is based on three assumptions: First, it is possible to systematically identify a set of meaningful features in the data, described as Codes. Second, the data have local structures, described as Conversations. Third, an important feature of the data is how Codes are connected to one another within Conversations [24,25,26].

ENA was originally designed to address challenges in learning analytics, however, the method is not limited to the analysis of learning data. ENA, for example, has been used in the analysis of (a) operative performance of surgery trainees’ during a simulated procedure [52]; (b) gaze coordination during collaborative work [53]; (c) communication among health care teams [54,55]. 

The key assumption of the method is that the structure of data connections is the most important aspect of the analysis. Thus, ENA is an appropriate analytic method for any context in which there is a meaningful structure of connections. ENA is a useful technique for modeling the eye-tracking data because it can model the connections (here, transitions) between the different AOIs as they occur while participants solve graph-based problems. 

In our study, the ENA algorithm uses a moving window to construct a network model for each line in the data (here, fixation points), pointing out how Codes in the current line are associated to Codes that occur within the recent temporal context [56], defined as three lines (the current line plus the two previous ones) within a given Conversation. The resulting networks were aggregated for all lines, and for every unit of analysis in the model. We aggregated networks using a weighted summation where the networks for a given line reflect the log of the product of each pair of Codes.

The ENA networks were visualized (see Section 4, Figures 5–12) using network graphs in which nodes corresponding to the Codes and the edges width reflect the relative frequency of co-occurrence, or associations, between two Codes. The result is two coordinated representations for each unit of analysis: (1) a plotted point, representing the location of that unit’s network in the low-dimensional projected space, and (2) a weighted network graph. The locations of the network graph nodes are fixed, and those locations are determined by an optimization routine minimizing the difference between the plotted points and their corresponding network centroids. This projected space and co-registration of network graphs enables the positions of the network graph nodes—and the associations they define—can be used to interpret the dimensions of the projected space and explain the positions of plotted points in the space.

In this study, ENA was used to compare correct and incorrect solvers based on their plotted point locations, individual networks, mean plotted point locations, and mean networks, which average the association weights across individual networks. ENA also allowed us to compare the networks using network difference graphs. These graphs are calculated by subtracting the weight of each association in one network from the corresponding associations in another.

To examine the transitions between the AOIs and to contrast them between correct and incorrect solvers, we defined the units of analysis as all the lines of data related with the individual score subsets of the participants. Our ENA model included the AOIs as Codes. We defined conversations as all the lines of data related with individual score subsets of the participants. 

All eight ENA models (one for each graph task) had co-registrations correlations from 0.94 to 0.99 (Pearson) and from 0.93 to 0.99 (Spearman) for the first dimension, and co-registration correlations from 0.97 to 0.99 (Pearson) and from 0.98 to 0.99 (Spearman) for the second. These measures indicate a strong goodness of fit between the visualizations and the original models.

## 4. Results

### 4.1. Comparison of Fixation Frequencies between Correct and Incorrect Solvers

To answer RQ1, we added up the number of fixations for correct and incorrect solvers for each task and calculated the relative fixation frequencies for each AOI. To compare the fixation frequencies between correct and incorrect solvers, we used *t*-tests for independent samples for each AOI within a task. Table 3 shows the proportion of fixations that the students with correct and incorrect solutions had in each of the eight tasks. The Fisher criterion of *p* = 0.05 was applied to determine the significance level (For the full results of the *t*-test look at Appendix A Table A1).

By looking at the amount of correct and incorrect solvers for the Area under the curve related problems we can identify that for all these problems with the exception of PhyAreaQual there were more participants that provided an incorrect answer. In the case of the problems related to the slope of the graph results show inverted results. For all slope related problems with the exception of EcSlopeQuant there were more participants that provided a correct answer. Results also show that the number of correct answers for qualitative problems was higher than for the quantitative ones.

Since the main focus of all tasks was placed on analyzing a graph, as expected, all students fixated on each task’s graph most often. When looking at the frequencies of fixation on the AOI_Question, incorrect solvers showed significantly more fixations only in tasks where a calculation of the area under a curve (AreaQuant) was required. While there were no significant differences in the fixations on the *y*-axis label, in tasks in which the slope of a curve had to be interpreted verbally, *x*-axis labels were fixated on significantly more often by incorrect solvers. While, again, no differences were found regarding the AOI_graph in five out of eight tasks, correct solvers fixated on the attractor more often. The analysis of the individual axes (*x*-axis and *y*-axis) showed only isolated, unsystematic findings in terms of differences between the two groups of students. In contrast, the fixation frequencies on the distractors differed more notably. While students with wrong solutions fixated on the distractors for the SlopeQuant and SlopeQual in economics tasks more often, correct solvers for qualitative physics tasks fixated more often on the attractor. This finding may be related to a domain effect, i.e., students found it more difficult to understand the meaning of distractors in a foreign domain (physics) than in a familiar domain (economics).

**Figure 3:**
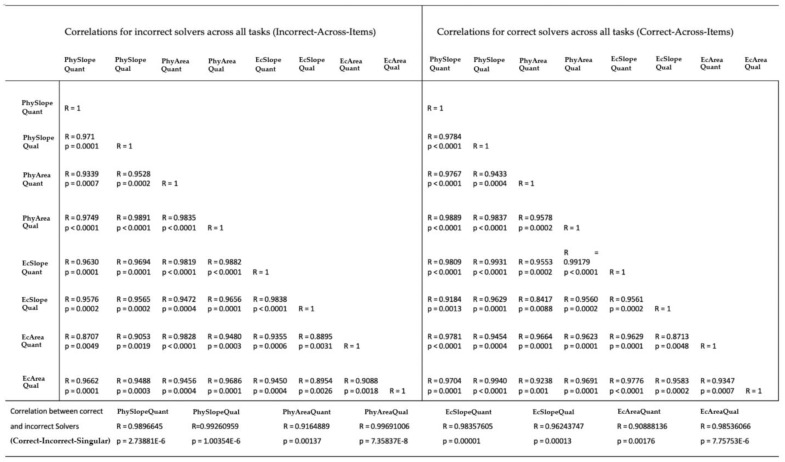
Correlations for % of AOI fixation. Top Left: Correlations for incorrect solvers across all tasks (Incorrect-Across-Items). Top Right: Correlations for correct solvers across all tasks (Correct-Across-Items). Bottom: Correlations between correct and incorrect solvers for each individual task (Correct-Incorrect-Singular).

To analyze how stable the gaze patterns of incorrect and correct solvers were between the individual tasks, we calculated the correlation of fixation frequencies for these groups between the tasks based on the AOIs. We examined the average proportion of incorrect solvers’ fixations on each of the AOIs for each of the graph tasks (Incorrect-Across-Items) and calculated a Pearson correlation to investigate commonalities of incorrect solving performances among the graph problems. There was a significant strong positive association for the proportion of fixations spent on certain AOIs among all graph tasks for both incorrect and correct solvers (Figure 3).

We also calculated a Pearson correlation between incorrect and correct responses for each individual graph problem (Correct-Incorrect-Singular). There was a significant strong positive association for the proportion of fixations on the AOIs between correct and incorrect solvers for each individual task (Figure 3 Bottom). 

Next, we compared the coefficient of the correlations from Correct-Incorrect-Singular with Incorrect-Across-Items and Correct-Across-Items. With the exception of the tasks EcAreaQuant and PhyAreaQuant, the correlation coefficients for Correct-Incorrect-Singular were higher than the average correlation coefficients from Incorrect-Multiple and Correct-Multiple. Therefore, when investigating correct and incorrect strategies to solve graph problems by examining the proportion of AOI fixations, each graph problem should be analyzed individually.

### 4.2. Gaze Transition Comparison between Correct and Incorrect Responses

To answer RQ 2, the frequencies of occurrence of the individual transitions were compared, followed by an analysis of the interrelationships of the transition frequencies using ENA. To analyze gaze transitions, we looked at the direction of the transitions, as participants often looked back and forth between AOIs (e.g., between Question and Graph), in which case these transitions could be counted as distinct types of transitions.

**Figure 4:**
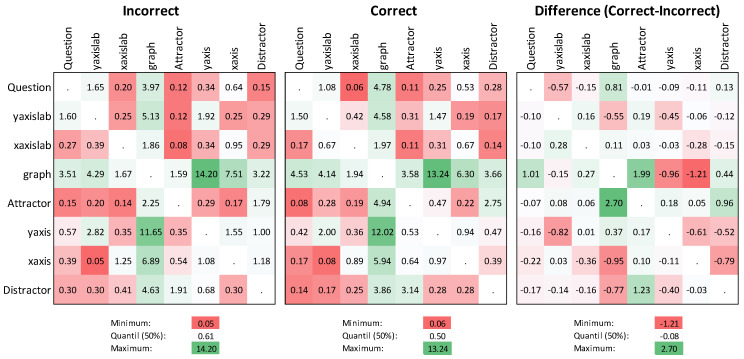
Proportion of the transitions (%) for incorrect (top) and correct solvers (bottom). The color illustrates the difference plot of relative transition frequency differentiated by the 50% quantile (white, incorrect solver: 0.61, correct solver: 0.50) from very rare (intense red) to very frequent (intense green).

Figure 4 shows the percentages of the types of transitions that occur during the processing of the graph tasks among incorrect (left) and correct solvers (middle), and the difference between correct and incorrect solvers (right). The figure shows the distribution of the percentual transition frequencies aggregated over all tasks. The values below the diagonal represent the proportion of transitions that are directed from the row labels to the column labels. Above the diagonal, the proportions of the transition that start from the columns and end in the rows are shown. The cells of the matrix were divided into quantiles. First, the 50% quantile of each matrix was determined, which is shown in the figure in a neutral white background. The minimum and maximum of each matrix were determined, and highlighted in red or green, respectively. All values between the minimum and the 50% quantile become brighter and less red the closer they are to the quantile. All values above the 50% quantile are highlighted in green, and become progressively whiter the closer they are to the quantile. The different colors of the individual cells in the table highlight the distance from the quantile.

For example, for incorrect solvers, the proportion of transitions that start at the AOI_yaxislab and end at the AOI_Question is 1.6% of all transitions. Conversely, for correct solvers, 1.65% of all transitions start from AOI_Question and end at AOI_yaxislab. The transitions from the graph to the *y*-axis and vice versa are the most common types of transitions for both incorrect and correct solvers (dark green) at almost 25%. The two transition types with the lowest probability of occurrence are the transitions from the *x*-axis to AOI_yaxislab (0.05%) and from AOI_xaxislab to the attractor (0.08%). For correct solvers, the least probable transitions are the transitions from the AOI_Question to AOI_xaxislab (0.05%) and, with an identical frequency of occurrence (0.08%), the transitions from the attractor to the question and from AOI_xaxislab to AOI_yaxislab. This representation, however, only shows which share a certain transition has in relation to all transitions in the response process of correct and incorrect solvers. To show the differences between correct and incorrect solvers, it is necessary to look at the differences in the individual transitions. To depict this, the difference table was created. In this matrix, the values are shaded in a deeper green the more the percentage of correct solvers exceeds the percentage of incorrect solvers by, and vice versa. The differences in the transition between attractor and graph in particular are more pronounced the correct solvers. The differences in the transitions between axes and graph more pronounced among the incorrect solvers. However, the relative proportions of the transitions have to be considered, and are less frequent from the graph to the axes than the transitions between attractor and graph.

However, these percentages do not necessarily express the importance of a transition. On the one hand, a small percentage can occur because a particular transition plays a smaller role in the solver’s response process. On the other hand, it is also possible that this type of transition occurs less frequently because the AOIs are more distal to each other or differ in size, so that the specificity-sensitivity problem of the AOI definition must be considered [34]. In any case, a more precise analysis would require a weighting of the transitions and a consideration of the fixations within an AOI to identify differences in the response processes.

In a first step, we conducted a Pearson correlation to further examine the symmetrical distribution between the diagonals (Figure 4). We found a strong correlation between the direction of all of the transitions, indicating that the direction of a transition is less important than the frequency of the transitions and their relation to the task solution behavior (Table 4).

Since we found a symmetrical transition structure, we added up the number of transitions for each type of transition to analyze differences in the frequency of transitions between incorrect and correct solvers for each of the eight tasks. We did not differentiate between the direction of transition (e.g., transitions from question to graph and from graph to question were considered the same transition type); therefore, in our analysis, we only differentiated between 28 transition types.

The differences between the two groups were calculated for each of the 28 transition types and for each task using a *t*-test with independent samples, as the students solved each task either correctly or incorrectly. All 184 individual response processes were included in the analyses (23 students × 8 tasks).

Table 5 shows the *t*-values and the significances; *t*-values with a positive sign indicate that the incorrect solver had more transitions and negative signs indicate that the correct solver had more transitions. The values and significance of the individual transition types vary greatly between the eight tasks. Incorrect solvers had significantly more transitions between distractors and the *y*-axis than correct solvers in the AreaQualPhys task, and in the AreaQuantFin task, correct solvers again show a significantly higher proportion of transitions between attractors and distractors than incorrect solvers.

Although individual differences were identified, no uniform picture emerged regarding the differences in the transition frequencies of individual transition types across tasks. The isolated consideration of the individual transitions did not take into account the complexity of and connections between the transitions, without which students would not be able to solve the task. For example, in the solution process, some students might compare the attractor and graph several times while others might compare the attractor to the distractors more often. To take into account the complexity of transaction patterns, ENA was used in the next step.

**Figure 5:**
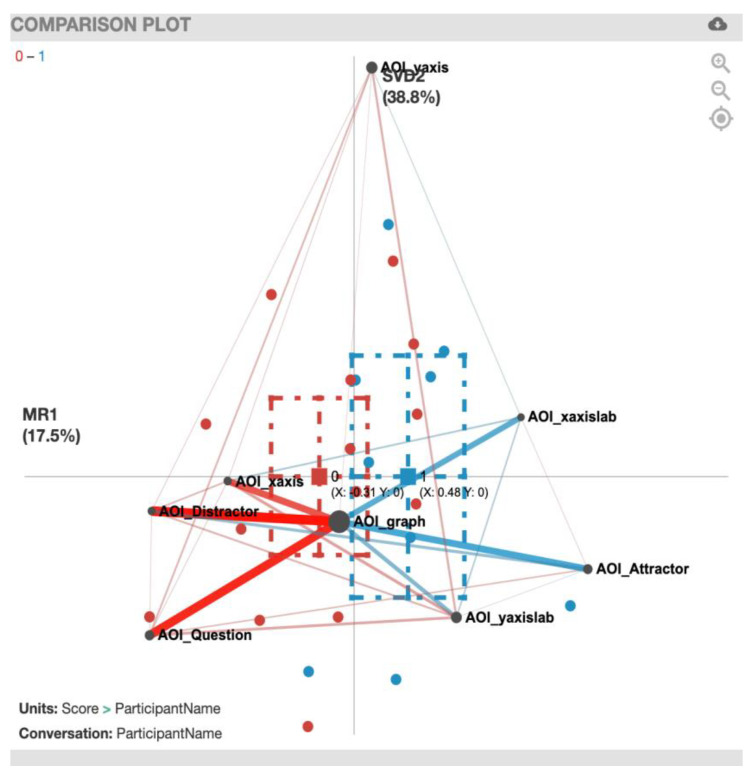
EcAreaQual transitions with incorrect solvers (red) and correct solvers (blue). MR1 presents a variance of 17.5% and SDV2 presents a variance of 38.8%.

**Figure 6:**
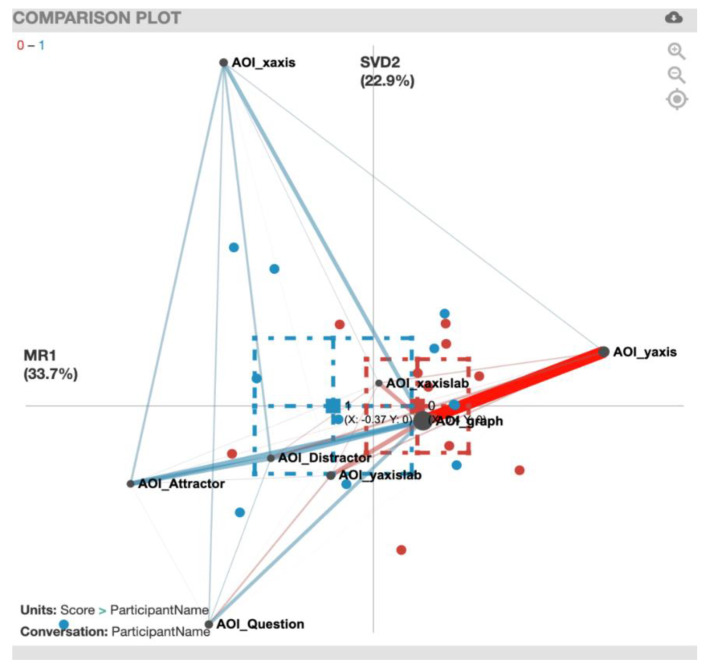
PhyAreaQual transitions with incorrect solvers (red) and correct solvers (blue).

**Figure 7:**
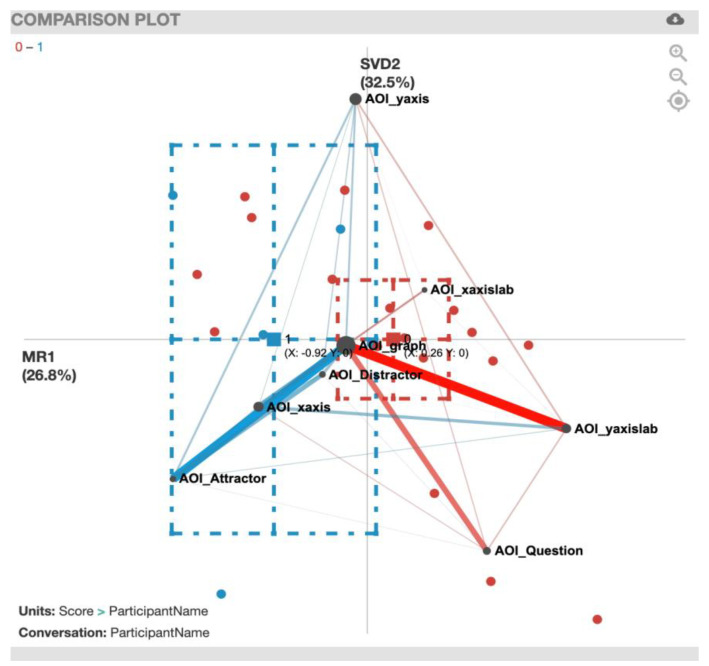
EcAreaQuant transitions with incorrect solvers (red) and correct solvers (blue).

**Figure 8:**
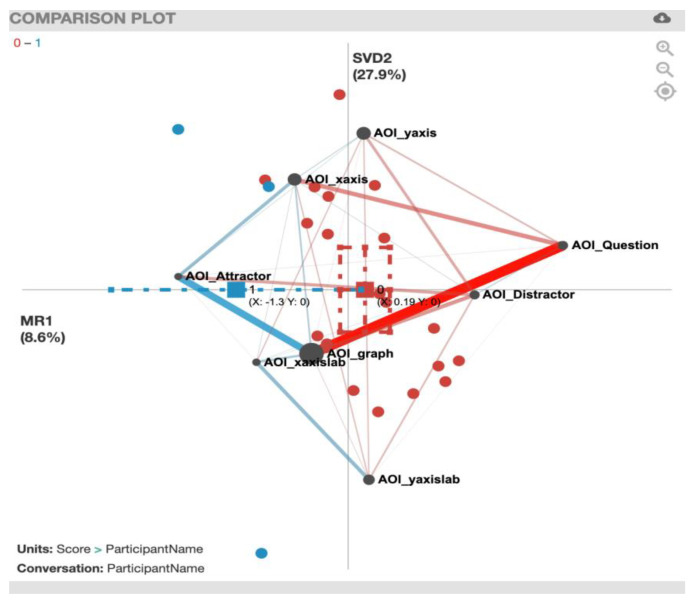
PhyAreaQuant transitions with incorrect solvers (red) and correct solvers (blue).

**Figure 9:**
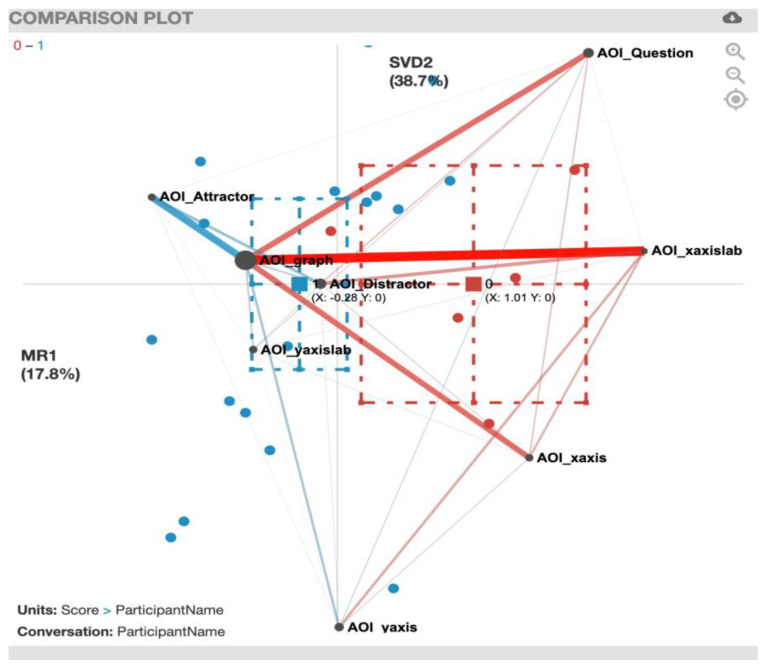
EcSlopeQual transitions with incorrect solvers (red) and correct solvers (blue).

**Figure 10:**
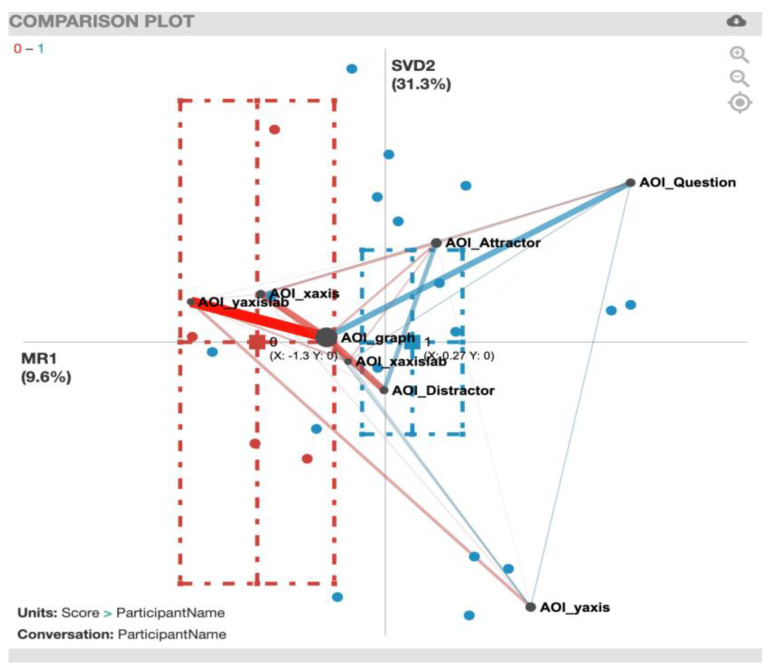
PhySlopeQual transitions with incorrect solvers (red) and correct solvers (blue).

**Figure 11:**
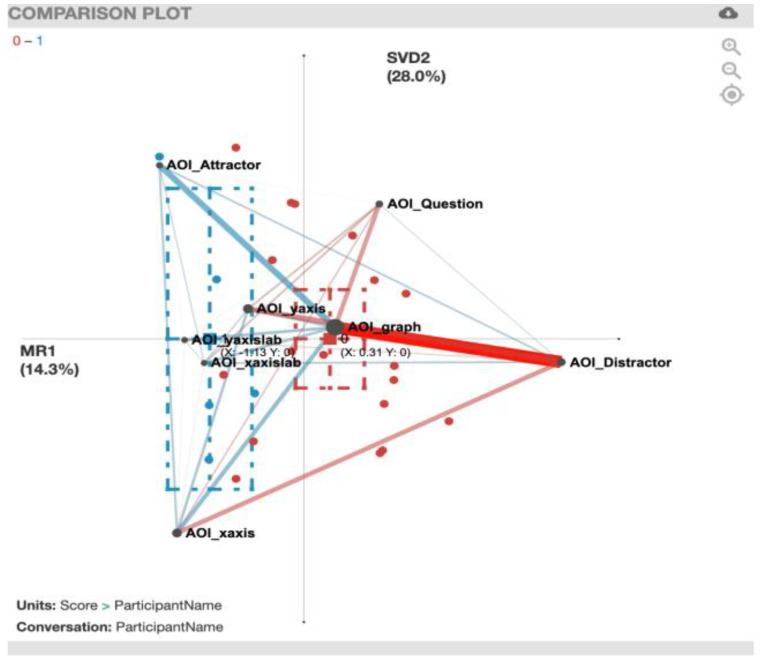
EcSlopeQuant transitions with incorrect solvers (red) and correct solvers (blue).

**Figure 12:**
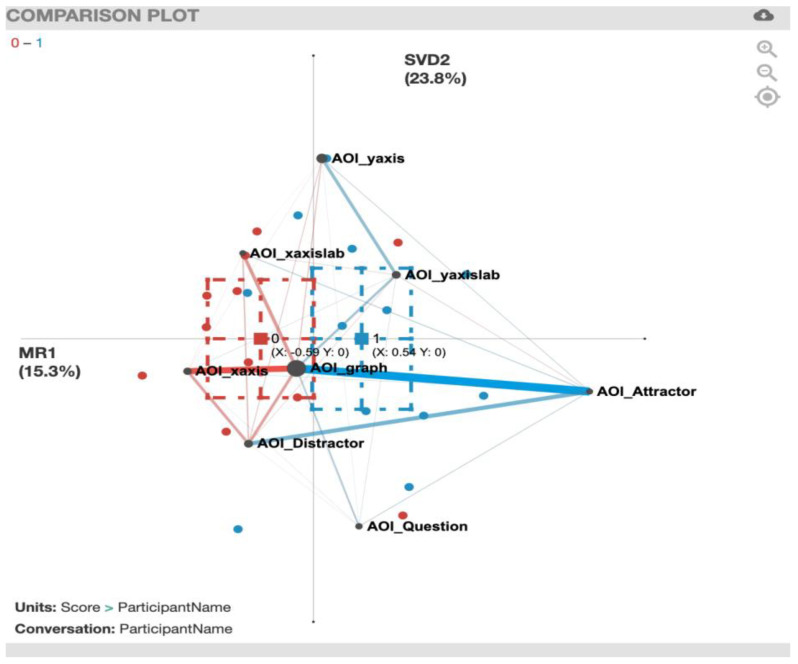
PhySlopeQuant transitions with incorrect solvers (red) and correct solvers (blue).

We created ENA comparison plots for correct *vs*. incorrect solvers showing the AOI associations for the eight tasks (Figure 5, Figure 6, Figure 7, Figure 8, Figure 9, Figure 10, Figure 11 and Figure 12). To plot the multidimensional graph created by ENA into a two-dimensional representation for the *x*-axis, we used MR1, which aligns the centroids of the compared groups. Hence, the y-value displayed for the correct and incorrect solvers is always equal to 0 in all the figures. For the *y*-axis, we used the singular value decomposition (SVD2) for the AOIs that presented the biggest variance for each of the tasks.

The comparison plot for the EcAreaQual task (Figure 5) presents the differences in weighted transition frequencies between incorrect and correct solvers and shows the so-termed ‘AOI_graph’ in the center of the network graph, since the graph was most frequently involved in all transitions. The plot also shows how AOIs such as AOI_xaxis, AOI_Distractor, and AOI_Question are located on the left side of the graph, meaning that they are closely related to the centroid of the incorrect solvers. In contrast, AOIs such as AOI_xaxislab, AOI_Attractor, and AOI_yaxislab are located on the right side of the graph, meaning that they have a closer relation with the centroid for the correct solvers.

When comparing the centroids of the transition patterns of incorrect solvers and correct solvers using a two-sample *t*-test, while assuming unequal variance along the two-dimensional structure (*x*-axis and *y*-axis of the network graph), significant differences between incorrect solvers (mean=-0. 31, SD = 0.74, N = 14) and correct solvers (mean = 0.48, SD = 0.65, N = 9; t(18.86) = −2.68, *p* = 0.01, Cohen’s d = 1.11) become evident along the *x*-axis.

In terms of the associations among the different AOIs, the red lines in the plot indicate the correlations that were most common among incorrect solvers, and the blue lines represent the correlations that were most common among correct solvers. The thicker the lines, the stronger the correlations. For a summary of the strong gaze transition correlations, see Table 6.

For the ENA plot of the PhyAreaQual task (Figure 6), along the *x*-axis, a two-sample *t*-test assuming unequal variance (mean = 0.40, SD = 0.69, N = 11) indicated no statistically significant differences at the alpha level = 0.05 (mean = −0.37, SD = 1.13, N = 12; t(18.50) = −1.99, *p* = 0.06, Cohen’s d = 0.81). The plot shows that there was a particularly strong correlation of gaze transition between the AOI_graph and AOI_yaxis for the incorrect solvers (red). For a summary of the strong gaze transition correlations, see Table 6.

For the ENA plot of the EcAreaQuant task (Figure 7), along the *x*-axis, a two-sample *t*-test assuming unequal variance (mean = 0.26, SD = 1.10, N = 18) showed statistically significant differences at the alpha level = 0.05 (mean = −0.92, SD = 0.81, N = 5; t(8.61) = −2.64, *p* = 0.03, Cohen’s d = 1.12). The plot shows a strong correlation of gaze transition between the AOI_graph and AOI_yaxislab for the incorrect solvers (red) and a strong gaze transition correlation between AOI_graph and AOI_Attractor for correct solvers (blue). For a summary of the strong gaze transition correlations, see Table 6.

For the ENA plot of the PhyAreaQuant task (Figure 8), along the *x*-axis, a two-sample *t*-test assuming unequal variance (mean = 0.19, SD = 0.61, N = 20) indicated statistically significant differences at the alpha level = 0.05 (mean = −1.30, SD = 0.58, N = 3; t(2.72) = −4.10, p = 0.03, Cohen’s d = 2.44). In contrast to the previously presented plots, this plot presents strong gaze transition correlations that are not connected to the AOI_graph, for example AOI_Question and AOI_xaxis for incorrect solvers (red). For a summary of the strong gaze transition correlations, see Table 6.

For the ENA plot of the EcSlopeQual task (Figure 9), along the *x*-axis, a two-sample *t*-test assuming unequal variance (mean = 1.01, SD = 0.67, N = 5) indicated statistically significant differences at the alpha level = 0.05 (mean = −0.28, SD = 0.71, N = 18; t(6.71) = −3.76, *p* = 0.01, Cohen’s d = 1.84). Similar to the previous plots, this plot presents strong gaze transition correlations between the AOI_graph and AOI_Attractor for correct solvers (blue). For a summary of the strong gaze transition correlations, see Table 6.

For the ENA plot of the PhySlopeQual task (Figure 10), along the *x*-axis, a two-sample *t*-test assuming unequal variance (mean = −1.30, SD = 0.49, N = 4) indicated statistically significant differences at the alpha level = 0.05 (mean = 0.27, SD = 1.06, N = 19; t(10.10) = −4.56, *p* = 0.00, Cohen’s d = 1.58). In this plot, the strong gaze transition correlation between AOI_Attractor and AOI_Distractor among the correct solvers (blue) is notable. For a summary of the strong gaze transition correlations, see Table 6.

For the ENA plot of the EcSlopeQuant task (Figure 11), along the *x*-axis, a two-sample *t*-test assuming unequal variance (mean = 0.31, SD = 0.83, N = 18) indicated statistically significant differences at the alpha level = 0.05 (mean = −1.13, SD = 0.41, N = 5; t(14.14) = 5.40, *p* = 0.00, Cohen’s d = 1.88). Besides a strong gaze transition correlation between the AOI_graph and AOI_Distractor for the incorrect solvers (red), there is a gaze correlation between AOI_Distractor and AOI_Attractor for the correct solvers (blue). For a summary of the strong gaze transition correlations, see Table 6.

For the ENA plot of the PhySlopeQuant task (Figure 12), along the *x*-axis, a two-sample *t*-test assuming unequal variance (mean = −0.59, SD = 0.88, N = 11) indicated statistically significant differences at the alpha level = 0.05 (mean = 0.54, SD = 0.87, N = 12; t(20.77) = 3.08, *p* = 0.01, Cohen’s d = 1.29). In this plot, the most noticeable gaze transition correlation is the one between AOI_graph and AOI_Attractor for the correct solvers (blue). For a summary of the strong gaze transition correlations, see Table 6.

To synthesize the ENA results, Table 6 shows the significant interrelations between gaze transitions across the graph problems, grouped by correct and incorrect solvers. Overall, for 7 of the 8 tasks, correct solvers had a significant correlation to gaze transitions between AOI_graph and AOI_Attractor as well as between AOI_Attractor and AOI_Distractor. Incorrect solvers had a significant correlation to gaze transitions between AOI_graph and AOI_Question as well as between AOI_graph and AOI_Distractor for 5 of the 8 graph problems.

## 5. Discussion and Conclusions

In this study, we examined eye-tracking-based fixation and transition data from first-year economics students while solving graph-based tasks to investigate the differences and commonalities between correct and incorrect solution patterns. For an in-depth investigation of the transitional eye-tracking metric on the basis of correct and incorrect solutions, we used ENA. With ENA, we chose a holistic approach that considers the compensatory and selective characteristics of eye movements more explicitly than previous studies that only included fixation frequencies or transitions between individual AOIs.

First, we examined the fixation frequencies of correct and incorrect solvers in the AOIs (RQ1).

*RQ1: How do correct and incorrect solvers differ in their proportion of fixations on AOIs of graph understanding tasks?*

When examining each of the tasks individually, correct and incorrect solvers showed significant differences on the percentage of their fixation frequencies on at least one of the AOIs for each of the eight tasks. Three of the tasks showed significant differences in the percentage of fixation frequencies for three AOIs; two tasks showed significant differences in two AOIs; and three tasks showed significant differences in only one of the AOIs. It became evident that individual analyses of graph tasks are necessary since they can each place different demands on the learner, which are also reflected in the fixation frequencies.

Concurring with Madsen et al. [12], who show that students who solve a task correctly dwell longer on selected areas of a graph, our findings show, as expected, that correct solvers also look at these areas more frequently. For tasks in which the fixation frequency on the AOI attractor varies significantly, the correct solvers always looked at the attractor more often than the incorrect solvers. Assuming that the fixation frequency correlates with the dwell time in AOIs [34], the present study’s results are comparable to the findings of other studies [1,3,12]. In accordance with the findings [1,12] that the dwell time on the attractor as a solution-relevant factor correlates with solving the task correctly, our results show that correct solvers fixate on the attractor more frequently.

In addition, there were differences in terms of the concepts presented in the tasks. For example, in three out of four tasks in which a qualitative assessment of the slope or area under the curve was required, the *x*-axis or the *x*-axis label was looked at more frequently by incorrect solvers. As Susac et al. [3] and Madsen et al. [12] pointed out, this reflects an ineffective solution behavior since the focus on the abscissa was irrelevant in these tasks, as it was mainly the graph itself or distances on the *y*-axis that had to be considered.

In the calculation tasks for areas (AreaQuant), which were evidently more difficult to solve than slope tasks [1,3,55], incorrect solvers fixated on the question more often. The task questions contain relevant information about the task scenario, context and problem; thus, processing this information already demanded a lot of cognitive resources from the students.

Moreover, correct solvers focused on distractors more frequently in two out of four qualitative tasks. This can also be explained by the difference between qualitative and quantitative tasks. In calculations based on formulas, such as the linear slope or the area under the curve, students may determine the result of a task in an almost linear solution process, without even having seen the answer options. Thus, when the result of a task was immediately determined this way, the other answer options might have received less attention. This was different for the qualitative tasks, where the information given in the task scenarios and the questions must be processed, compared and integrated with the answer options given in each task [32].

The findings indicate which different response processes with regard to the graphic concepts and mathematical requirements presented therein the tasks elicit in students. Thus, we examined whether the significant differences identified in the individual tasks could be generalized across all tasks. Within individual tasks, correct and incorrect solvers shared a greater proportion of fixations on the AOIs than correct solvers and incorrect solvers did across the problems. This supports the assumption that tasks with the graph concept of area under a curve are more difficult and address different cognitive challenges for students than slope tasks and therefore require differentiated treatment [1,3,14].

The finding pertaining to RQ1 illustrates that the proportion of eye fixations can be used to identify correct and incorrect graph task solving strategies only for individual tasks. However, the findings cannot be generalized for all types of graph tasks. It cannot be ruled out that the graph problems were not similar enough to find generalizable strategies across them. To generalize across tasks, the differences in graph concepts and the mathematical and context-specific requirements must be considered. The number of participants and number of tasks used in this study pose additional limitations in terms of generalization of results. By extending the number of participants in further research, we expect to identify clearer incorrect and correct solving strategies for single items, and larger differences across the tasks. However, the sample presented here exceeds the samples of other studies using standardized tests tasks, which comprise 6 and 14 students respectively [23].

In comparison to many recent graph understanding studies using eye-tracking [1,3,14], which focus merely on the dwell times in the domains of physics and economics, our study adds the analysis of fixations and a holistic analysis of transitions. To our knowledge, this is the first study investigating these metrics to assess the understanding of economics tasks of economics students. Therefore, in a second step, we examined the following RQ:

*RQ2: How do correct and incorrect solvers differ in their transitions on different AOIs of graph understanding tasks?*

Transitions are a powerful eye-tracking metric to analyze gaze shifts between, for example, different web page elements [57], distances in air traffic observation [58], use of software [59] or image-text integration of assessments [43]. Previous studies on graph understanding [23,41,44] between different groups (here, correct and incorrect solvers) showed that certain transitions occur more often for one group than for others. By analyzing the individual transitions among the AOIs, we first identified a strong correlation for the direction of these transitions. Thus, when identifying properties of graph problem solution behavior, the frequency of transitions between two AOIs is more important than their respective direction and there are more transitions between adjacent AOIs than distant AOIs [34,44,45]. The most frequent transition, between the graph and the *y*-axis, addresses a gaze movement that is in line with expectations for the natural movement of the eye, which can execute horizontal gaze shifts more easily than vertical ones [34]. At the same time, it is typical for the task solving process since a frequent comparison between *y*-axis segments and the curve is important for estimating slopes and areas below a curve. Other horizontal transitions like comparisons between AOI_yaxis and AOI_yaxislab were less frequent, indicating that they were less important for the response process than expected. Overall, the findings show no uniform picture of the differences in the frequencies of the individual transition types across tasks and for correct and incorrect solvers.

We argue that the transitions among AOIs have a temporal context, i.e., a transition is influenced by the total number and type of all transitions that occur within a response process. To examine the transitions in different contexts, we conducted an ENA for each of the tasks. The ENA allowed us to identify significant differences in the AOI transitions between correct and incorrect solvers for seven of the eight tasks. These findings revealed that there are important connections between the AOIs that need to be identified and processed by learners to correctly solve graph tasks.

The use of ENA for the analysis of eye-tracking data allowed us to gain insights that were not obtainable by analyzing only the immediate transitions among AOIs. For example, by using a moving window of three transitions, our ENA findings show how correct solvers had strong transitions between AOI_graph and AOI_Attractor and as well as strong transitions between AOI_Attractor and AOI_Distractor. This indicates that correct solvers were not just moving their gaze between AOI_Attractor and AOI_Distractor more than incorrect solvers. The moving window of transitions used for this study allowed us to identify that correct solvers compared AOI_Attractor to AOI_Distractor by looking at different parts of the problem and then returning their gaze to the possible solutions. Furthermore, in comparison to other network analysis methods [43], ENA offers the possibility of a simple inferential statistical comparison between two or more groups by calculating the centroid with a 95% confidence interval. This makes it easier to analyze a complex interdependent structure, as it is the case with transitions.

The transition association between AOI_graph and AOI_Attractor reflects a complementary and at the same time parallel visual and textual representation of a graph problem, which has to be integrated by the participants to find the solution. Thus, this text-image integration of identical phenomena [29,39] seems to be required for the understanding of economic graph problems. The transition associations of correct solvers between attractor and distractor(s) is also in line with expectations, as correct solvers tend to verify that their solution is correct by comparing it with the distractors. Studies on verbal data in response process analyses using the think-aloud method show that incorrect solvers did not perform a systematic comparison and simply selected any solution, i.e., a guessing behavior [43]. The eye movement data of the present study suggest similar findings as well.

The think-aloud method is also a useful addition to eye-tracking studies as it can provide semantic meaning to fixations and transitions. At the same time, it can also influence eye-tracking, for instance if the fixation time increases during simultaneous think-alouds due to the higher cognitive load, thus potentially leading to asynchronous or delayed verbalization, and gaze shifts [60]. In the present study, the tasks are quite challenging for the test participants, so that a retrospective analysis of verbal data could be useful to explore the meaning of the participants’ eye movements in further studies.

For incorrect solvers, we identified particularly strong transitions between AOI_graph and AOI_Question, and AOI_graph and AOI_xaxis. The first transition indicates that incorrect solvers have a greater need to reconcile information between the question and the graph, for example to re-explore the question for more information as to what exactly they are supposed to look for in the graph, or to compensate for misunderstandings based on erroneous situational models. In line with the findings of Tsai et al. (2012) [23], the respondents often did not know how to proceed to solve the task and therefore searched for additional information in the question. The second transition is less relevant for solving a task correctly, since, for instance, for the solution of qualitative gradient tasks the steepness of the graphs was considered or for the estimation of the areas under a curve the distances between the curve along the *y*-axis had to be compared. Students with a less pronounced understanding of graphs are more likely to linger on irrelevant areas such as the *x*-axis of a graph [1,12,22]. However, our results suggest that transitions are a quite significant additional indicator, complementary to the dwell time on the axes and the gaze shifts from the axes to the graph.

Since the understanding of an independent variable on the *x*-axis and the dependent variable on the *y*-axis taught in school is often reversed in economics, for instance, by presenting the price as an independent variable on the *y*-axis and the quantity of a product demanded or supplied on the *x*-axis, new problems in graph understanding become apparent, which should be more strongly addressed by transition research in the future. For example, it would be possible to swap the axes in diagrams or to investigate the specifics of economic graph understanding with inverse graphs. In our study, in which a comparison of graph understanding was made between physics and economics graphs, such a question was not considered. On the basis of our findings, however, this is necessary for a subsequent study to address the significance of axes especially in economics, for which still no standardized test exists, as opposed to, for example, the TUG-K in physics [8].

Once more, the number of participants and the number and kind of tasks observed present a limitation to generalizing the results regarding the difference in AOI transition patterns between correct and incorrect solvers (RQ2).

Apparently, vertical eye movements describe the decisive difference between incorrect and correct solvers. The most frequent transition, between graph and *y*-axis, is often (schematically) executed by both groups and cannot be characteristic for a difference between the groups. Vertical eye movements require more effort than horizontal eye movements and are performed more selectively, for example to develop a certain idea or to search for specific information. The size and positioning of the AOIs are often the subject of numerous discussions about the precision of the measurement of relevant fixations [34]. In the present study, the AOIs were defined in such a way that a sufficient distance to the actual text or graph allows for as many relevant fixations as possible to be captured. If the AOIs were too small, too few fixations may be recorded, which would hinder a statistically robust analysis. If the range was too large, fixations may be detected [35]. Further specifications are necessary for subsequent investigations, for example pertaining to reading behavior measured by saccades and fixations within an AOI. Thus, it is quite plausible to assume that students with a greater reading ability will better understand the task and be better able to solve it. This assumption should be investigated in further studies. Due to the assumption that the individual AOIs are assigned different importance by the solver over time, some studies suggest a time-sequential analysis of the transitions for individual time intervals [42,53,59,61]. For example, in the present case, it is possible that AOI_Question is considered by some students, depending on their solution strategy, only at the beginning but not at the end of the task solving process. Following on from the present study, future research should examine whether changes in transition frequencies over time occur.

Regardless of the limitations mentioned above, our study shows that sensors, in this particular case eye-trackers, can be used to identify correct and incorrect graph problem solving strategies, and therefore provide educational insights. Particularly, ENA can be applied for the analysis of eye-tracking data in graph problem solving. Our findings suggest directions for the development of educational interventions designed to foster graph understanding and graph problem solving. For example, learners can be instructed to re-evaluate all the answers before arriving at a solution, even when they think they know the correct one. Our results also suggest that Intelligent Tutoring Systems (ITSs) can be enhanced with eye-tracking methods to improve instruction by reminding learners to check all options before providing an answer when identifying weak gaze transition connections between attractor and distractors. Moreover, when identifying strong gaze transition connections between the graph and the question, ITSs can automatically ask learners if they require a clarification of the problem. While eye-trackers currently are not broadly available devices, they are becoming increasingly more affordable. Furthermore, high resolution webcams integrated in personal computers can already perform eye-tracking functions to a certain extent. Thus, we foresee a real possibility of having ITSs enhanced with eye-tracking capabilities in the near future.

To conclude, eye-tracking combined with ENA in the context of learning analytics have the potential to support students in learning how to solve graph problems, and hence better understand graphs, a significant skill in both everyday life and in various domains.

## Figures and Tables

**Table 1:** Sample extract of the analyzed data.

Case	Participant	Media	Record.T.	Start	End	Fix.Index	Process	AOI_Quest.	AOI_Yaxisl.	AOI_Graph	…
1	ae04rcVe	AreaQualfin	370589	12:25:22.46	12:25:22.65	1112	AOI_Question	1	0	0	…
1	ae04rcVe	AreaQualfin	370781	12:25:22.65	12:25:22.90	1113	AOI_Question	1	0	0	…
1	ae04rcVe	AreaQualfin	371031	12:25:22.90	12:25:23.06	1114	AOI_Question	1	0	0	…
1	ae04rcVe	AreaQualfin	371189	12:25:23.06	12:25:23.14	1115	AOI_graph	0	0	1	…
1	ae04rcVe	AreaQualfin	371272	12:25:23.14	12:25:23.38	1116	AOI_graph	0	0	1	…
1	ae04rcVe	AreaQualfin	371514	12:25:23.38	12:25:23.62	1117	AOI_Question	1	0	0	…
1	ae04rcVe	AreaQualfin	371756	12:25:23.62	12:25:23.77	1118	AOI_Question	1	0	0	…
…	…	…	…	…	…	…	…	…	…	…	…

**Table 2:** Sum of transitions per single AOI.

Question	Yaxislab	Xaxislab	Graph	Attractor	Yaxis	Xaxis	Distractor
1328	1753	793	6987	1192	3004	1815	1539

**Table 3:** Average number of fixations of correct and incorrect solvers for each task in percent (significant differences in bold; * *p* > 0.5; ** *p* > 0.01).

AOI	ECAreaQual		PhyAreaQual		EcAreaQuant		PhyAreaQuant		EcSlopeQual		PhylopeQual		EcSlopeQuant		PhySlopeQuant	
	✘	✔	✘	✔	✘	✔	✘	✔	✘	✔	✘	✔	✘	✔	✘	✔
AOI_Question	30.4%	25.5%	23.5%	21.6%	**36.2%**	19.9% *	**28.9%**	13.5% *	21.8%	30.2%	20.4%	24.2%	25.3%	24.1%	15.6%	18.4%
AOI_yaxislab	6.8%	7.7%	5.7%	5.5%	11.7%	7.6%	6.8%	9.3%	2.6%	2.1%	3.9%	2.8%	3.4%	2.9%	6.9%	8.0%
AOI_xaxislab	1.5%	1.7%	2.4%	1.7%	1.0%	0.6%	1.7%	4.1%	**2.4%**	0.6% **	2.0%	1.1%	2.6%	2.8%	4.1%	2.2%
AOI_graph	50.3%	53.9%	54.9%	53.7%	52.5%	64.0%	50.8%	64.8%	49.6%	46.1%	57.4%	56.5%	51.5%	55.6%	62.5%	59.7%
AOI_Attractor	8.3%	11.9%	5.4%	5.1%	**1.5%**	3.7% *	**1.9%**	5.8% *	**1.5%**	6.7% *	10.2%	8.2%	**3.6%**	7.5% *	**2.8%**	6.6% **
AOI_yaxis	5.9%	6.6%	8.9%	7.8%	**10.4%**	20.1% *	9.2%	9.5%	2.7%	3.4%	5.3%	4.2%	9.6%	8.8%	9.9%	10.0%
AOI_xaxis	**2.9%**	1.5% *	5.3%	6.7%	6.2%	7.4%	8.3%	12.5%	3.1%	1.8%	**4.9%**	2.4% *	7.1%	7.9%	6.1%	4.0%
AOI_Distractor	8.8%	5.2%	**9.0%**	11.7% *	3.2%	5.6%	7.6%	5.2%	**17.5%**	12.0% *	**3.0%**	4.2% *	**14.2%**	7.7% *	13.8%	9.2%
Number of solvers	14	9	11	12	18	5	20	3	5	18	4	19	18	5	11	12

**Table 4:** Correlations between the direction of transitions for incorrect and correct solvers.

	Incorrect Solvers		Correct Solvers	
	Correlation R	*p*-Value	Correlation R	*p*-Value
AOI_Question	0.98826	4.01 × 10^−1^	0.99264	9.90 × 10^−2^
AOI_yaxislab	0.96159	0.00014	0.92150	0.00114
AOI_xaxislab	0.96236	0.00013	0.96873	0.00007
AOI_graph	0.98055	0.00002	0.98529	7.87 × 10^−1^
AOI_Attractor	0.93042	0.00080	0.94725	0.00035
AOI_yaxis	0.99497	3.17 × 10^−2^	0.99882	4.10 × 10^−4^
AOI_xaxis	0.98343	0.00001	0.99320	7.83 × 10^−2^
AOI_Distractor	0.90852	0.00179	0.99200	1.27 × 10^−1^

**Table 5:** *T*-test results showing the differences in transition frequencies between correct and incorrect solvers per task for each of the 28 transition types. Negative values indicate differences that favor correct solvers (Bold = significant result, * *p* < 0.05, ** *p* < 0.01).

	AreaQualFin	AreaQualPhys	AreaQuantFin	AreaQuantPhys	SlopeQualFin	SlopeQualPhys	SlopeQuantFin	SlopeQuantPhys
AOI_AttractorAOI_Distractor	−0.763	−0.133	−2.155 *	0.440	−1.129	0.309	−0.633	0.626
AOI_AttractorAOI_graph	−1.943	1.817	−2.371 *	−2.418 *	−2.315 *	1.645	−1.118	−1.809
AOI_AttractorAOI_question	−2.280 *	1.914	−0.755	-	-	1.518	−0.131	−0.518
AOI_AttractorAOI_xaxis	−0.195	−1.130	−0.961	−0.641	-	1.873	0.091	−1.915
AOI_AttractorAOI_xaxislab	−0.442	−0.518	0.518	0.552	-	3.370 **	−1.348	0.062
AOI_AttractorAOI_yaxis	−0.693	1.929	−1.552	−1.000	−0.755	0.757	−1.166	0.589
AOI_AttractorAOI_yaxislab	−1.254	−1.915	0.812	−1.000	−1.844	−0.655	−1.000	0.749
AOI_DistractorAOI_graph	1.294	0.176	−0.885	−0.039	−0.513	2.473	1.258	2.361 *
AOI_DistractorAOI_question	0.211	0.077	−1.100	−0.601	1.201	−0.450	−0.901	0.677
AOI_DistractorAOI_xaxis	1.749	−0.309	−0.916	−0.863	0.916	−0.450	0.825	2.362 *
AOI_DistractorAOI_xaxislab	0.795	1.060	−0.131	2.517 *	1.968	−0.450	−1.230	1.763
AOI_DistractorAOI_yaxis	1.472	2.257 *	−1.149	0.823	−0.102	1.525	1.223	1.785
AOI_DistractorAOI_yaxislab	1.385	2.084	−0.641	0.828	0.166	−0.450	2.715 *	1.454
AOI_graphAOI_question	−0.274	0.176	−0.351	2.325 *	1.694	2.396	0.210	0.334
AOI_graphAOI_xaxis	0.314	1.066	−2.284 *	−0.769	0.667	1.313	−0.859	2.380 *
AOI_graphAOI_xaxislab	−1.308	1.512	−1.551	−0.395	0.901	1.070	−0.178	1.824
AOI_graphAOI_yaxis	−0.707	1.811	−0.974	−0.193	−0.583	0.653	−0.297	2.029
AOI_graphAOI_yaxislab	−1.833	1.588	−0.076	−0.168	−0.596	3.642	0.052	1.523
AOI_questionAOI_xaxis	1.546	0.380	−0.797	0.812	−0.518	0.951	0.493	0.086
AOI_questionAOI_xaxislab	0.324	0.594	-	−0.757	−0.518	−0.655	1.314	1.000
AOI_questionAOI_yaxis	−1.060	1.112	0.215	3.249 **	−2.204 *	−0.450	0.961	1.191
AOI_questionAOI_yaxislab	0.921	2.390 *	0.268	−0.546	−0.377	1.072	0.709	1.269
AOI_xaxisAOI_xaxislab	−1.023	1.129	1.330	−0.849	1.804	1.633	0.337	0.915
AOI_xaxisAOI_yaxis	−0.055	−0.547	−1.421	1.326	0.166	1.217	−0.266	1.510
AOI_xaxisAOI_yaxislab	0.211	0.481	−0.033	−2.671 *	-	-	2.204 *	−1.393
AOI_xaxislabAOI_yaxis	−0.615	1.234	−0.166	0.552	0.904	−0.772	−0.828	2.506 *
AOI_xaxislabAOI_yaxislab	−1.284	0.362	−0.502	−0.734	−1.000	0.691	−1.188	0.156
AOI_yaxisAOI_yaxislab	0.230	1.899	−0.404	−0.279	0.777	0.856	0.458	1.172

**Table 6:** Strong associations between gaze transitions across the tasks and correct vs. incorrect solvers. (Sum of strong associations across tasks in bold).

	EcArea Qual		PhyArea Qual		EcArea Quant		PhyArea Quant		EcSlope Qual		PhySlope Qual		EcSlope Quant		PhySlope Quant		Total	
**Strong Transition Associations**	✔	✘	✔	✘	✔	✘	✔	✘	✔	✘	✔	✘	✔	✘	✔	✘	**✔**	**✘**
AOI_graph and AOI_Attractor	1		1		1		1		1			1	1		1		**7**	**1**
AOI_graph and AOI_yaxislab	1			1		1						1			1		**2**	**3**
AOI_graph and AOI_xaxislab	1			1						1						1	**1**	**3**
AOI_graph and AOI_xaxis		1	1							1		1	1			1	**2**	**4**
AOI_graph and AOI_yaxis				1					1		1			1			**2**	**2**
AOI_graph and AOI_Distractor		1						1				1		1		1	**0**	**5**
AOI_graph and AOI_Question		1	1			1		1		1	1			1			**2**	**5**
AOI_Attractor and AOI_Distractor	1		1		1			1	1		1		1		1		**7**	**1**
AOI_xaxis and AOI_yaxis													1				**1**	**0**
AOI_xaxislab and AOI_yaxislab	1						1			1							**2**	**1**
AOI_xaxislab and AOI_Distractor										1							**0**	**1**
AOI_xaxis and AOI_Attractor			1				1										**2**	**0**
AOI_yaxislab and AOI_xaxis					1												**1**	**0**
AOI_yaxislab and AOI_yaxis						1						1			1		**1**	**2**
AOI_yaxis and AOI_Attractor					1												**1**	**0**
AOI_Distractor and AOI_yaxis								1									**0**	**1**
AOI_Question and AOI_xaxis			1					1				1					**1**	**2**
AOI_Question and AOI_yaxis								1									**0**	**1**
AOI_Question and AOI_yaxislab						1											**0**	**1**

## Appendix A

**Table A1:** Average number of fixations of correct and incorrect solvers for each task including values of the *T*-tests (significant differences in bold).

AOI	ECAreaQual		PhyAreaQual		EcAreaQuant		PhyAreaQuant		EcSlopeQual		PhylopeQual		EcSlopeQuant		PhySlopeQuant	
	incorrect	correct	incorrect	correct	incorrect	correct	incorrect	correct	incorrect	correct	incorrect	correct	incorrect	correct	incorrect	correct
AOI_Question	30.4%	25.5%	23.5%	21.6%	**36.2%**	**19.9%**	**28.9%**	**13.5%**	21.8%	30.2%	20.4%	24.2%	25.3%	24.1%	15.6%	18.4%
	t(21) = 1.05; *p* = 0.3		t(21) = 0.65; *p* = 0.52		**t(21) = 2.22; *p* = 0.04**		**t(21) = 1.86; *p* = 0.04**		t(21) = −1.6; *p* = 0.13		t(21) = −0.7; *p* = 0.49		t(21) = 0.18; *p* = 0.86		t(21) = 0.61; *p* = 0.55	
AOI_yaxislab	6.8%	7.7%	5.7%	5.5%	11.7%	7.6%	6.8%	9.3%	2.6%	2.1%	3.9%	2.8%	3.4%	2.9%	6.9%	8.0%
	t(21) = −0.7; *p* = 0.49		t(21) = 0.32; *p* = 0.75		t(21) = 1.24; *p* = 0.23		t(21) = −0.87; *p* = 0.23		t(21) = 0.69; *p* = 0.55		t(21) = 0.48; *p* = 0.63		t(21) = 0.49; *p* = 0.63		t(21) = 0.55; *p* = 0.59	
AOI_xaxislab	1.5%	1.7%	2.4%	1.7%	1.0%	0.6%	1.7%	4.1%	**2.4%**	**0.6%**	2.0%	1.1%	2.6%	2.8%	4.1%	2.2%
	t(21) = 0.39; *p* = 0.7		t(21) = 1.36; *p* = 0.19		t(21) = 0.66; *p* = 0.52		t(21) = −1.58; *p* = 0.52		**t(21) = 3.82; *p* < 0.01**		t(21) = 1.29; *p* = 0.21		t(21) = 0.31; *p* = 0.76		t(21) = 1.42; *p* = 0.17	
AOI_graph	50.3%	53.9%	54.9%	53.7%	52.5%	64.0%	50.8%	64.8%	49.6%	46.1%	57.4%	56.5%	51.5%	55.6%	62.5%	59.7%
	t(21) = −0.64; *p* = 0.52		t(21) = 0.38; *p* = 0.7		t(21) = −1.65; *p* = 0.11		t(21) = −1.67; *p* = 0.11		t(21) = 0.50; *p* = 0.62		t(21) = 0.14; *p* = 0.89		t(21) = −0.58; *p* = 0.56		t(21) = 0.56; *p* = 0.58	
AOI_Attractor	8.3%	11.9%	5.4%	5.1%	**1.5%**	**3.7%**	**1.9%**	**5.8%**	**1.5%**	**6.7%**	10.2%	8.2%	**3.6%**	**7.5%**	**2.8%**	**6.6%**
	t(21) = −2.04; *p* = 0.05		t(21) = 0.4; *p* = 0.69		**t(21) = −2.6; *p* = 0.02**		**t(21) = −3.4; *p* = 0.03**		**t(21) = −2.53; *p* = 0.02**		t(21) = 0.87; *p* = 0.39		**t(21) = −2.3; *p* = 0.03**		**t(21) = −3.7; *p* < 0.01**	
AOI_yaxis	5.9%	6.6%	8.9%	7.8%	**10.4%**	**20.1%**	9.2%	9.5%	2.7%	3.4%	5.3%	4.2%	9.6%	8.8%	9.9%	10.0%
	t(21) = 0.27; *p* = 0.79		t(21) = 0.88; *p* = 0.39		**t(21) = −2.7; *p* = 0.01**		t(21) = −0.101; *p* = 0.9		t(21) = −0.35; *p* = 0.73		t(21) = 0.66; *p* = 0.52		t(21) = −0.34; *p* = 0.7		t(21) = −0.05; *p* = 0.96	
AOI_xaxis	**2.9%**	**1.5%**	5.3%	6.7%	6.2%	7.4%	8.3%	12.5%	3.1%	1.8%	**4.9%**	**2.4%**	7.1%	7.9%	6.1%	4.0%
	**t(21) = 2.01; *p* = 0.049**		t(21) = −1.44; *p* = 0.17		t(21) = −0.80; *p* = 0.43		t(21) = −1.73; *p* = 0.09		t(21) = 1.57; *p* = 0.13		**t(21) = 2.36; *p* = 0.03**		t(21) = −0.32; *p* = 0.8		t(21) = 1.72; *p* = 0.09	
AOI_Distractor	8.8%	5.2%	**9.0%**	**11.7%**	3.2%	5.6%	7.6%	5.2%	**17.5%**	**12.0%**	**3.0%**	**4.2%**	**14.2%**	**7.7%**	13.8%	9.2%
	t(21) = 1.75; *p* = 0.09		**t(21) = 2.19; *p* = 0.04**		t(21) = −1.47; *p* = 0.16		t(21) = 0.94; *p* = 0.36		**t(21) = 2.74; *p* = 0.01**		**t(21) = 2.42; *p* = 0.03**		**t(21) = 2.6; *p* = 0.02**		t(21) = 2.5; *p* = 0.019	
